# A Tidy and Healthful Home

**Published:** 1890-10

**Authors:** 


### A TIDY AND HEALTHFUL HOME.

Looking at some "cluttered-up" households, we are reminded of the philanthropic Mrs. Jellyby's family, who, if they had possessed the entire space of St. Paul's for a habitation, would only have found it to be so much more room to be untidy in. People who are untidy in a small house, are likely to be still more untidy in a big one. Few of us can always achieve that comfortable doctrine of a place for everything and everything in its place. The first part of the saying is about as far as a good many of us get. But there is no question of its being the only rule for comfortable housekeeping. Take a small sitting-room, distribute about it two or three newspapers, a box of toys, a work-basket, a few unmended stockings, and perhaps a hat or coat, and what a miserably untidy place it looks. Nor is it right for the house mother to go about picking up after the careless ones. Let each member of the household learn that things must always be bestowed in their proper places, without trusting to the one tireless worker to straighten things up. The boys who always expect "mother" to do their tidying up, grow into the men who must be waited on so constantly by their wives. Nor is it only the boys who need a lecture in this respect. How many girls we know who slam down their hats and jackets on removing them, without a thought of laying them away. How often the best frock is carelessly put aside instead of being shaken out and put by carefully. And then they wonder that their clothes so soon look shabby. A well-cared-for garment will outlast two that are just slammed around, and next to getting good clothes, comes taking care of them.

